# microRNA‐365 attenuated intervertebral disc degeneration through modulating nucleus pulposus cell apoptosis and extracellular matrix degradation by targeting EFNA3

**DOI:** 10.1111/jcmm.18054

**Published:** 2023-11-27

**Authors:** Chao Jiang, Youjun Liu, Weigong Zhao, Yimin Yang, Zhiwei Ren, Xiaohui Wang, Dingjun Hao, Heng Du, Si Yin

**Affiliations:** ^1^ Department of Orthopedic Surgery The First Affiliated Hospital of Xi'an Jiaotong University Xi'an China; ^2^ Department of Developmental Genetics Max Planck Institute for Heart and Lung Research Bad Nauheim Germany

**Keywords:** apoptosis, ephrin‐A3, extracellular matrix, intervertebral disc degeneration, miR‐365

## Abstract

This present study is aimed to investigate the role of microRNA‐365 (miR‐365) in the development of intervertebral disc degeneration (IDD). Nucleus pulposus (NP) cells were transfected by miR‐365 mimic and miR‐365 inhibitor, respectively. Concomitantly, the transfection efficiency and the expression level of miRNA were detected by quantitative reverse transcription polymerase chain reaction (qRT‐PCR). Meanwhile, NP cells apoptosis was measured through propidium iodide (PI)‐AnnexinV‐fluorescein isothiocyanate (FITC) apoptosis detection kit. Subsequently, immunofluorescence (IF) staining was performed to assess the expression of collagen II, aggrecan and matrix metalloproteinase 13 (MMP‐13). In addition, bioinformatic prediction and Luciferase reporter assay were used to reveal the target gene of miR‐365. Finally, we isolated the primary NP cells from rats and injected NP‐miR‐365 in rat IDD models. The results showed that overexpression of miR‐365 could effectively inhibit NP cells apoptosis and MMP‐13 expression and upregulate the expression of collagen II and aggrecan. Conversely, suppression of miR‐365 enhanced NP cell apoptosis and elevated MMP‐13 expression, but decreased the expression of collagen II and aggrecan. Moreover, the further data demonstrated that miR‐365 mediated NP cell degradation through targeting ephrin‐A3 (EFNA3). In addition, the cells apoptosis and catabolic markers were increased in NP cells when EFNA3 upregulated. More importantly, the vivo data supported that miR‐365‐NP cells injection ameliorated IDD in rats models. miR‐365 could alleviate the development of IDD by regulating NP cell apoptosis and ECM degradation, which is likely mediated by targeting EFNA3. Therefore, miR‐365 may be a promising therapeutic avenue for treatment IDD through EFNA3.

## 1 INTRODUCTION

The incidence of low back pain (LBP) has been increasing for many years, but few long‐term, effective curative treatments are available. Thus, this condition is a major public health problem worldwide. Although the mechanisms and aetiologies responsible for LBP are not still completely elucidated, intervertebral disc degeneration (IDD) is considered as one of the primary causes of LBP.^1^, ^2^, ^3^ In the early stages of IDD, there are no obvious signs or symptoms. However, it gradually becomes severe as an individual ages and results in spinal stenosis, spinal segmental instability, osteophyte formation, disc herniation and compression of the spinal cord and nerve root.^4^ Surgical intervention is an effective approach for patients suffering from IDD in the final stage.^5^ More importantly, Cheung et al.^6^ reported that 40% individuals under the age of 30 years suffered from IDD, which increased progressively to over 90% from 50 to 55 years of age. LBP makes the individuals suffering torment and increases economic and social burdens, especially in the health care field.^7^, ^8^ To the best of our knowledge, the normal intervertebral disc (IVD) in mammals is composed of three integral parts: the nucleus pulposus (NP) cells, annulus fibrosus (AF) and cartilage endplates.^4^, ^9^ Long‐term overloading, along with factors such as injury, smoking and ageing, can lead to IVD degeneration. This degeneration is characterized by progressive dysfunction and loss of disc cells, as well as metabolic disorders of the extracellular matrix (ECM).^10^ In normal IVD function, the anabolism and catabolism of the ECM are in dynamic equilibrium. However, when the homeostatic balance of the ECM is disrupted by various stimuli, the disc tends to undergo degeneration.^11^ This degenerative process involves multiple mechanisms, and the expression of cellular genes can be either enhanced or suppressed through epigenetic transcription or posttranscription. Numerous studies have demonstrated that various epigenetic modifications, including DNA methylation, histone modifications, RNA methylation (m^6^A) and mitochondrial quality control, play crucial roles in the pathogenesis of IVD degeneration.^10^, ^12^ NP cells are critical for sustaining the integrity of the IVD and function by producing the ECM, which is required for cell differentiation and proliferation.^9^ In addition, many other factors, including environmental and genetic factors (e.g. ageing, nutritional conditions, smoking, mechanical load),^13^, ^14^, ^15^ are involved in IDD via the regulation of NP cell activity. However, the mechanisms underlying IDD are not completely revealed.

MicroRNAs (miRNAs) are defined as a type of endogenous and small non‐coding RNA molecules with 20–22 nucleotides in length.^4^ Increasing studies have reported that miRNAs play crucial roles in regulating a range of biological functions, including cellular activities (e.g. proliferation, apoptosis and ECM metabolism) and tissue degeneration, by targeting the 3′‐untranslated region (3′‐UTR).^16^, ^17^, ^18^ Multiple studies have further reported that IDD is associated with the dysregulation of certain miRNAs, such as miR‐665,^19^ miR‐143‐5p,^20^ miR‐154^21^ and miR‐129‐5p,^22^ which play significant roles in the development of IDD. Recently, miR‐365, a member of the miRNAs family, has been found to affect the biology of various diseases, such as cancer,^23^, ^24^ rheumatoid arthritis (RA),^25^ and endplate chondrocyte degeneration.^26^ However, during IDD development, the pathophysiological mechanisms are relatively complex, and the functions of miR‐365 remain unknown.

In this study, we cultured human NP cells and transfected them with miR‐365 to determine whether miR‐365 mediates the progression of NP cell degradation. The results indicated that upregulation of miR‐365 expression could attenuate NP cell apoptosis and ECM catabolism. Importantly, further data demonstrated that miR‐365 mediated NP cell degradation through inhibiting EFNA3. In addition, the NP cell apoptosis and catabolic markers were significantly increased when EFNA3 expression was upregulated. Finally, we found that injection of miR‐365‐expressing NP cells injection ameliorated IDD in rats models through reducing cells apoptosis and ECM catabolism. Taken together, these findings demonstrated that miR‐365 could improve IDD by through targeting EFNA3.

## 2 MATERIALS AND METHODS

### 2.1 NP cell culture

Human NP cells were purchased from Procell (CP‐H097, China) and cultured in Dulbecco's Modified Eagle Medium/Ham's F‐12 (DMEM/F12, Hyclone, 30023.01B) supplemented with 10% foetal bovine serum (FBS, Hyclone, SH30070.03) and 1% penicillin–streptomycin (PS, Procell, PB180120). Subsequently, the NP cells were maintained at 37°C in a humidified atmosphere with 95% air and 5% CO_2_. Forty‐eight hours later, the 1/2 volume medium was replaced with fresh DEME/F12 medium containing the aforementioned components, and then the renewal of culture medium occurred each 3‐day intervals. When the confluence of NP cells reached 80%, the cells were detached and dissociated using trypsin (Hyclone, SH30042.01) and subcultured with the same DMEM/F12 as described previously herein for subsequent experiments. The morphology of NP cells was determined using Toluidine Blue O and Alcian Blue GX staining. In addition, an anti‐collagen II antibody (ab34712, 1:200, Abcam) was used to identify NP cells via immunocytochemistry.

### 2.2 NP cell transfection

Based on the experimental design, cells were divided into five groups as follows: control, inhibitor‐NC, inhibitor‐miR‐365, mimic‐NC and mimic‐miR‐365. Then, the inhibitor‐NC, miR‐365 inhibitor, mimic‐NC and miR‐365 mimic (GenePharma) were transfected into NP cells using Lipofectamine® 3000 Transfection Reagent (Invitrogen) according to the manufacturer's instructions. The sequences were as following:

inhibitor‐NC (5′‐CAGUACUUUUGUGUAGUACAA‐3′);

miR‐365 inhibitor (5′‐AUAAGGAUUUUUAGGGGCAUUA‐3′);

mimic‐NC (5′‐UUCUCCGAACGUGUCACGUTT‐3′, 5′‐ACGUGACACGUUCGGAGAATT‐3′);

miR‐365 mimic (5′‐UAAUGCCCCUAAAAAUCCUUAU‐3′, 5′‐AAGGAUUUUUAGGGGCAUUAUU‐3′).

### 2.3 Quantitative real‐time PCR

The total RNA was extracted to assess the efficiency of transfection by quantitative real‐time PCR (qRT‐PCR). After treatment, total RNA was harvested from NP cells using TRIzol reagent (Invitrogen) according to the manufacturer's guideline. Then, the expression of genes at mRNA level was determined by PrimeScript™ RT reagent Kit (Takara). The expression level of miR‐365 and EFNA3 were normalized to those of U6 and GAPDH, respectively. All experimental procedures were performed for three replicates. The primers were as following:

miR‐365 (F 5′‐CGTAATGCCCCTAAAAAT‐3′, R 5′‐GTGCAGGGTCCGAGGT‐3′);

EFNA3 (F 5′‐TCTCTGGGCTACGAGTTCCAC‐3′, R 5′‐CCTCAGACACTTCCAGTGCAG‐3′);

U6 (F 5′‐CTCGCTTCGGCAGCACA‐3′, R 5′‐AACGCTTCACGAATTTGCGT‐3′);

GAPDH (F 5′‐ACCACAGTCCATGCCATCAC‐3′, R 5′‐GTGAGGGAGATGCTCAGTGT‐3′).

### 2.4 NP cell apoptosis assay

At 48 h post‐transfection, the incidence of NP cell apoptosis was measured via flow cytometry (BD Accuri® C6) using an Annexin V‐FITC Apoptosis detection Kit (Invitrogen eBioscience, BMS500FI) based on the manufacturer's instruction. Briefly, the transfected cells were trypsinized, rinsed and resuspended. Concomitantly, the single cell suspension was incubated with the annexin V‐FITC (5 μL) and PI (5 μL) for 15 min in the dark at room temperature. Eventually, the flow cytometry was used to quantitatively assess the cells apoptosis. All experimental procedures were performed for three replicates, respectively.

### 2.5 Immunocytofluorescence staining

For immunocytofluorescence staining of each experimental group, NP cells (1 × 10^6^) were seeded on the coverslips. After treatment, the cells were rinsed for three times, fixed with 4% paraformaldehyde at room temperature for 15 min and permeabilized with Triton X‐100 in PBS for 10 min and incubated with 3% bovine serum albumin for 30 min. Then, the cells were incubated with primary antibodies overnight at 4°C. The primary antibodies for immunocytofluorescence staining included the following: anti‐collagen type II (15943‐1‐AP, 1:200, Proteintech), anti‐aggrecan (13880‐1‐AP, 1:300, Proteintech) and anti‐MMP13 (18165‐1‐AP, 1:100, Proteintech). Next, the coverslips were washed and mounted on the slides after incubating with the corresponding secondary antibodies. Subsequently, the images were obtained using confocal fluorescence microscope (Zeiss, LSM710). All experimental procedures were performed for three replicates, respectively.

### 2.6 Bioinformatic prediction and Luciferase reporter assay

Online software (TargetScan, Pictar, RNA22sites, PITAsites, miRanda) was used to detect the target genes of miR‐365. The candidate gene EFNA3 was found to be a potentially direct target gene of miR‐365. In addition, co‐transfection with pRL‐TK (40 ng) vector (Promega) containing EFNA3‐WT or EFNA3‐MUT and miR‐365‐3p or NC was conducted using Lipofectamine 3000 (Invitrogen) based on manufacturer's description. The experimental groups were divided as follows: miR‐365‐3p + EFNA3‐WT, miR‐365‐3p + EFNA3‐MUT, NC + EFNA3‐WT and NC + EFNA3‐MUT. After 48 h, the co‐transfected cells were harvested to detect firefly and renilla luciferase activities with Dual‐Luciferase® Reporter Assay System (Promega, E1910). All experimental procedures were performed for three replicates, respectively.

### 2.7 Western blotting assay

The NP cells were lysed in RIPA buffer for 30 min on ice for harvesting the total proteins after different treatments. Then, the concentration of protein was determined with bicinchoninic acid kit (BCA, Beyotime, P0010) following the manufacturer's description. The extracted proteins were separated using 10% sodium dodecyl sulphate‐polyacrylamide gel electrophoresis (SDS‐PAGE; Bio‐Rad, Mini Protean 3) and transferred to polyvinylidene fluoride (PVDF) membranes. The membrane was blocked with TBST containing 5% skim milk at room temperature for 90 min and incubated with primary antibody (anti‐EFNA3, 12480‐1‐AP, 1:1000, Proteintech; anti‐GAPDH, 200306‐7E4, 1:5000, Zen BioScience) at 4°C overnight. Subsequently, the membranes were co‐incubated with the corresponding secondary antibodies. The bands were detected and scanned by Bio‐Rad Image Lab System. All experimental procedures were performed for three replicates, respectively.

### 2.8 Culture and treatment of primary NP cells

Primary NP cells were obtained from the NP tissues of SD rats. Briefly, the intervertebral disc was dissected, removed the cartilaginous endplate, annulus fibrosus. Concomitantly, the remain tissue was minced and digested with collagenase II for 2 h. Digestion was terminated by DMEM/F12 containing 10% FBS and 1% PS, and the digested tissues were mechanically centrifuged (1000 rpm, 5 min). The dissociated cells were seeded into flask and cultured in 37°C incubator with 5% CO2. Half of medium was changed every 2–3 days until cells reached over 85% confluence. The purity of NP cells was identified by immunostaining with anti‐collagen II antibody.

NP cells were seeded in flask and transfected with Cy3‐miR‐365 mimic or Cy3‐mimic‐NC overexpressing lentivirus (GenePharma) according to the manufacturer's instructions. The transfection efficiency was measured by qRT‐PCR. The following primer sequences were used:

miR‐365 (F 5′‐GGGTAATGCCCCTAAAAAT‐3′, R 5′‐CAGTGCGTGTCGTGGAG‐3′);

U6 (F 5′‐GCTTCGGCAGCACATATACTAAAAT‐3′, R 5′‐CGCTTCACGAATTTGCGTGTCAT‐3′).

### 2.9 IDD models and injection

Sixty male SD rats were randomly divided into four groups as follows: sham group, rats only exposed intervertebral disc at Co6‐Co7 segment; IDD group, rats suffered from IDD surgery and were injected the same volume PBS; IDD + NP‐NC group, rats suffered from IDD surgery and were injected Cy3‐mimic‐NC NP cells; IDD + NP‐miR‐365 group, rats suffered from IDD surgery and were injected Cy3‐miR‐365 mimic NP cells.

The rat IDD models were developed according to previous description.^17^, ^27^ Briefly, the SD rats (12 weeks of age) were anesthetized with 1% sodium pentobarbital (60 mg/kg). To locate the disc position, a small skin incision was made from Co6 to Co8. Subsequently, a syringe needle (31G) was inserted vertically intervertebral disc at Co6‐Co7 segment, rotated 180°in the axial direction and kept for 10 s.

For cells injection, IDD models were treated with 20 μL Cy3‐miR‐365 mimic or Cy3‐mimic‐NC NP cells (1 × 10^6^) and the same volume PBS by local delivery at 1, 7 and 14 days post‐surgery based on previous descriptions,^17^, ^28^ respectively. Eight weeks after surgery, all rats were sacrificed, and the intervertebral discs tissues were collected for further evaluation.

### 2.10 Histological evaluation

Disc tissues were fixed with 4% paraformaldehyde for 72 h and decalcified in EDTA for 4 weeks. Next, the tissue was embedded in paraffin to prepare sections (4 μm). Thereafter, the sections were stained with haematoxylin and eosin staining kit (Beyotime, C0105M) and an improved Safranin O‐fast green cartilage staining kit (Solarbio, G1371) according to instructions. After staining, the sections were examined under microscope. The severity of disc degeneration was evaluated based on the modified histological grading system (Table 1).^17^ Specifically, this grading system is involved in 5 categories with scores from 0 (normal disc) to 15 points (severely degenerative disc), including morphology of the NP, cellularity of the NP, morphology of the AF, cellularity of the AF and border between the NP and AF.

**TABLE 1:** The modified histological grading system.^17^

Categories	Score 0	Score 2	Score 2	Score 3
Morphology of the NP	Round shape and the NP composes >75% of the disc area	Round shape and the NP composes 50%–75% of the disc area	Round shape and the NP composes 25%–50% of the disc area	Round shape and the NP composes <25% of the disc area
Cellularity of the NP	Stellar‐shaped cells with a proteoglycan matrix located at the periphery, evenly distributed	Coexistence of stellar and round cells, stellar cells are more than round cells	Mostly large, round cells separated by dense areas of proteoglycan matrix	Large, round cells separated by dense areas of proteoglycan matrix
Morphology of the AF	Collagen lamellae with no ruptures	Inward bulging, ruptured or serpentine fibres compose <25% of the AF	Inward bulging, ruptured or serpentine fibres compose 25%–50% of the AF	Inward bulging, ruptured or serpentine fibres compose >50% of the AF
Cellularity of the AF	Fibroblasts constitute >90% of the cells	Fibroblasts constitute >75%–90% of the cells	Intermediate	Chondrocytes constitute >75% of the cells
Border between the NP and AF	Without interruption	Minimal interruption	Moderate interruption	Severe interruption

### 2.11 Immunohistofluorescence and TUNEL staining

For immunohistofluorescence staining, the tissue sections were rinsed and blocked with 5% BSA. Subsequently, the sections were incubated with primary antibodies overnight at 4°C. The primary antibodies for immunohistofluorescence staining included the following: anti‐collagen type II (15943‐1‐AP, 1:100, Proteintech) anti‐aggrecan (13880‐1‐AP, 1:200, Proteintech) and anti‐MMP13 (18165‐1‐AP, 1:50, Proteintech). Sections were then rinsed and incubated with the corresponding secondary antibodies. Subsequently, images were obtainead from confocal fluorescence microscope (Zeiss, LSM710). To examine the cells apoptosis in vivo, TUNEL staining was conducted using a one‐step TUNEL apoptosis assay kit (Beyotime, Shanghai, C1088).

### 2.12 Statistical analysis

For all analyses, the values are expressed as representative mean ± standard deviation (SD) from at least three independent experiments. Statistical analyses were performed using SPSS 20.0 (IBM Corp). GraphPad Prism 6 (Graph Pad Software, Inc.) was employed to generate the plots. Statistical differences of groups were determined with the Student's *t*‐test or one‐way analysis of variance (anova). For all tests, significance was set at *p*‐value < 0.05 (two‐tailed).

## 3 RESULTS

### 3.1 Effect of miR‐365 overexpression on human NP cells apoptosis

**FIGURE 1:**
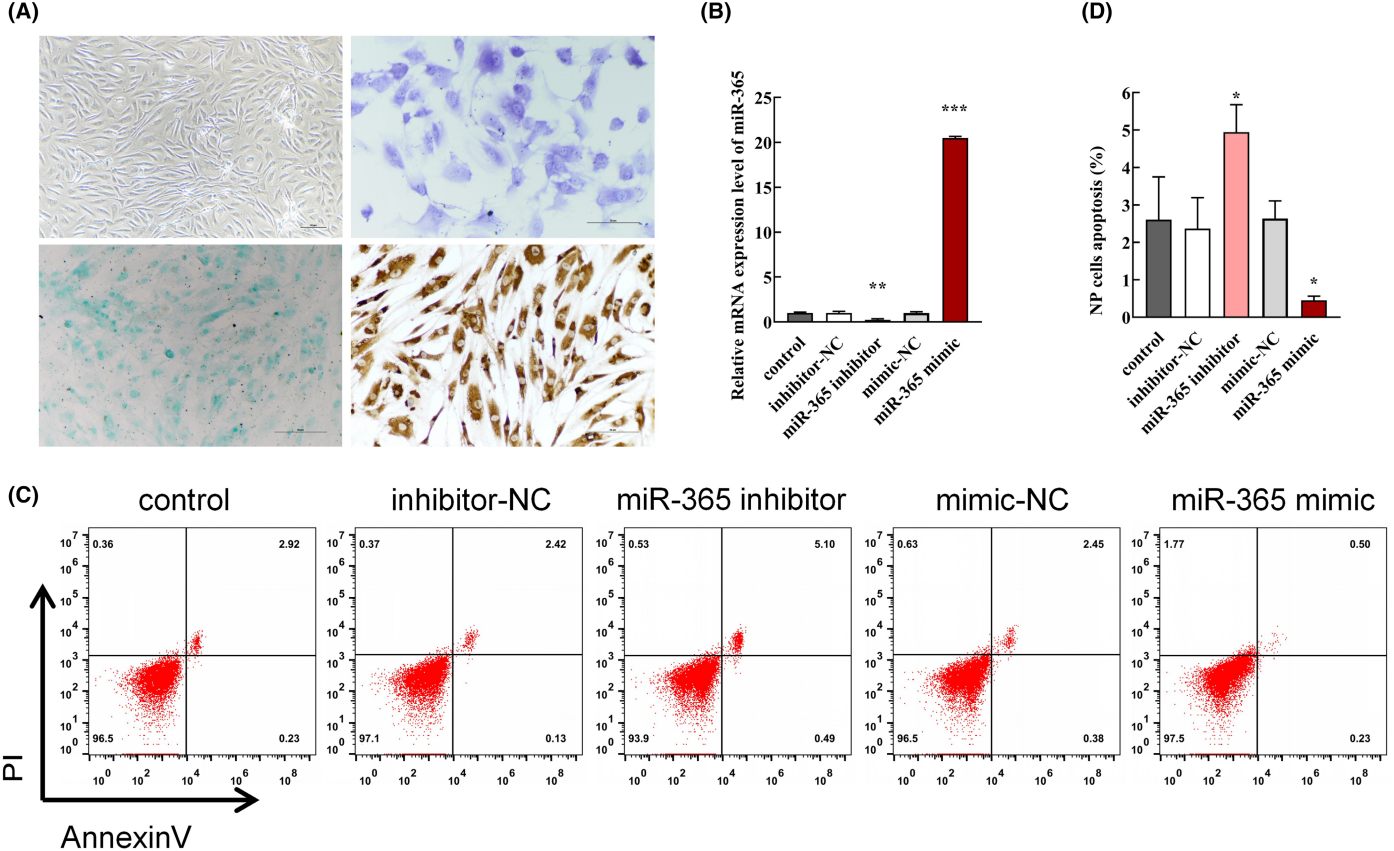
Effect of miR‐365 overexpression on NP cell apoptosis. (A) The morphology and structural properties of human NP cells (scale bar = 50 μm). (B) The expression of miR‐365 was analysed by qRT‐PCR after transfection with the miR‐365 mimic, mimic‐NC, miR‐365 inhibitor and inhibitor‐NC (*n* = 3/group, **p < 0.01, ***p < 0.001, compared with control group). (C) Detection of NP cell apoptosis via flow cytometry after transfection with miR‐365 mimic, mimic‐NC, miR‐365 inhibitor and inhibitor‐NC. (D) Quantification of NP cell apoptosis in each group (*n* = 3/group, *p < 0.05, compared with control group).

To perform the subsequent experiments, we first characterized and identified the human NP cells. Phase‐contrast, Toluidine Blue O and Alcian Blue GX staining showed that the large majority of cells exhibited a normal NP cell morphology and structure (Figure 1A). Notably, the NP cells displayed different morphological properties. Specifically, NP cells were elongated at a high cell density and flat at a small cell density. Immunocytochemistry assay further revealed that the majority of cells were collagen II‐positive.

**FIGURE 2:**
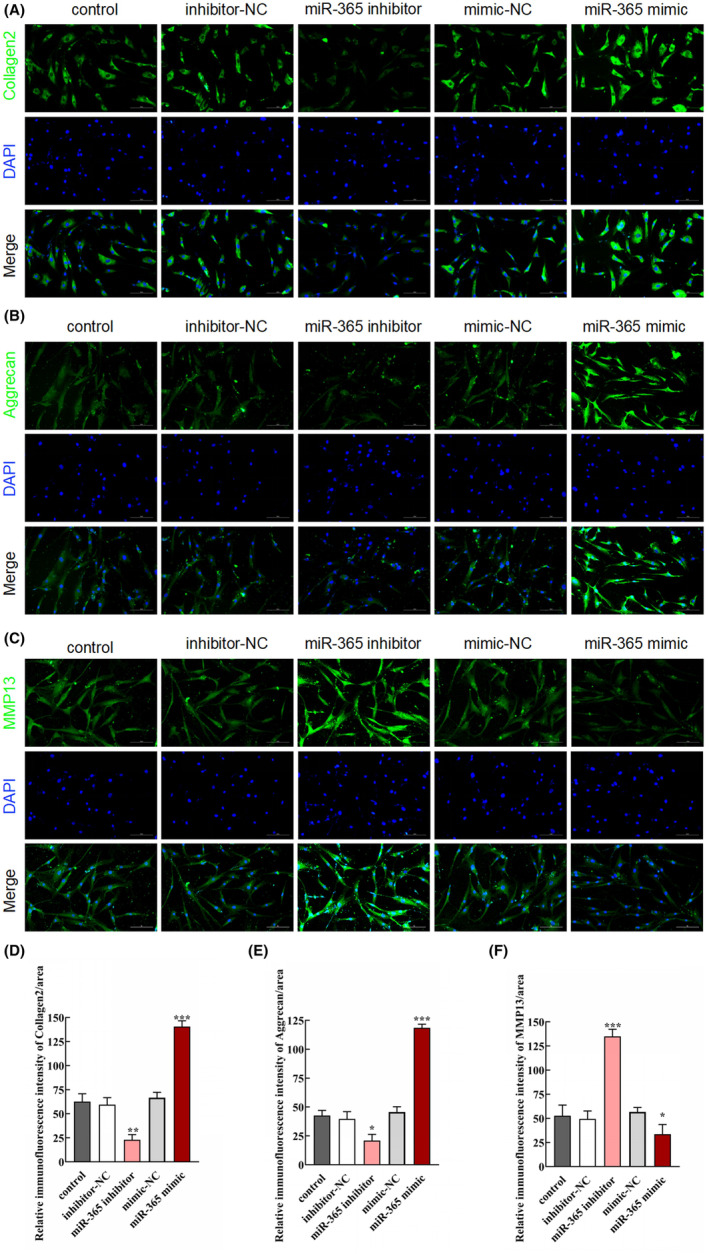
Effect of miR‐365 overexpression on ECM degradation. (A–C) Changes of collagen II, aggrecan and MMP‐13 expression were detected using immunofluorescence staining in each group (scale bar = 50 μm). (D–F) Quantification of the relative expression of collagen II, aggrecan and MMP‐13 in human NP cells after different treatments (*n* = 3/group, *p < 0.05, **p < 0.01 and ***p < 0.001, compared with control group).

To confirm the effect of miR‐365 overexpression on NP cells apoptosis, we performed qRT‐PCR in each group after NP cell treatment. The results showed that the expression level of miR‐365 was significantly elevated in the miR‐365 mimic group, indicating a highly efficient transfection (Figure 1B, ***p < 0.001, compared with control group). Moreover, flow cytometry analysis revealed that upregulation of miR‐365 significantly suppressed NP cell apoptosis (Figure 2C,D, *p < 0.05, compared with control group). Conversely, downregulation of miR‐365 with miR‐365 inhibitor enhanced NP cell apoptosis (Figure 2C,D, *p < 0.05, compared with control group).

### 3.2 Overexpression of miR‐365 inhibits ECM degradation

ECM is essential for normal NP cell functions and is mainly composed of collagen II and aggrecan. The overexpression of miR‐365 significantly increased the expression of collagen II (Figure 2A,D, ***p < 0.001, compared with control group) and aggrecan (Figure 2B,E, ***p < 0.001, compared with control group). On the contrary, inhibition of miR‐365 reduced expression of both collagen II (Figure 2A,D, **p < 0.01, compared with control group) and aggrecan (Figure 2B,E, *p < 0.05, compared with control group). In addition, matrix metalloproteinase 13 (MMP‐13) plays a critical role in ECM catabolism by mediating the degradation of ECM. Thus, we examined the effects of miR‐365 on MMP‐13 expression. The results displayed that overexpression of miR‐365 inhibited MMP‐13 expression, whereas suppression of miR‐365 promoted the expression level of MMP‐13 (Figure 2C,F, *p < 0.05, and ***p < 0.001, compared with control group).

### 3.3 miR‐365 directly associates with its target gene EFNA3

**FIGURE 3:**
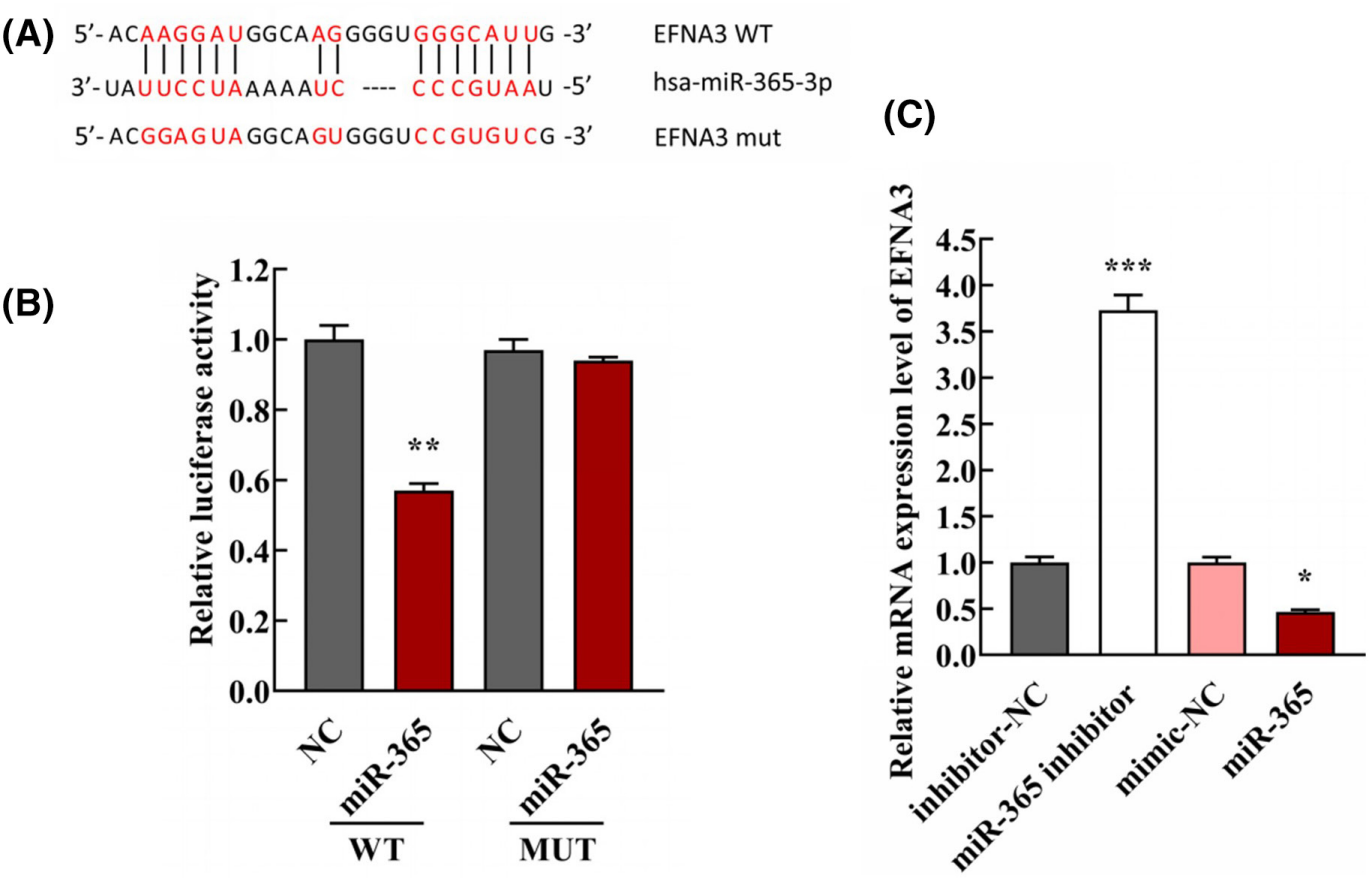
miR‐365 directly associates with the target gene EFNA3. (A) Bioinformatical analysis confirmed that miR‐365 contained complementary sequences for binding to wild‐type EFNA3. (B) Comparison of luciferase activity between wild‐type and mutant cells after transfection with miR‐365 mimic and mimic‐NC (*n* = 3/group, **p < 0.01, compared with NC group). (C) The expression level of EFNA3 mRNA was analysed by qRT‐PCR after transfection with the miR‐365 mimic, mimic‐NC, miR‐365 inhibitor and inhibitor‐NC (*n* = 3/group, *p < 0.05, ***p < 0.001, compared with inhibitor‐NC group).

Bioinformatic prediction suggested that miR‐365 contains complementary sequences that can bind the wild‐type 3′‐UTR of EFNA3 (Figure 3A). This potential target gene was further identified using luciferase reporter assay. The results demonstrated that upregulated expression of miR‐365 depressed the luciferase activity in the wild‐type group, but there was no significant alteration in the mutant‐type group (Figure 3B, **p < 0.01, compared with NC group). Moreover, the results demonstrated that the overexpression of miR‐365 inhibited the expression of EFNA3 (Figure 3C, *p < 0.05, compared with mimic‐NC group), whereas inhibition of miR‐365 enhanced EFNA3 expression (Figure 3C, ***p < 0.001, compared with inhibitor‐NC group). Thus, the evidences indicated that EFNA3 might be a target gene of miR‐365.

### 3.4 Upregulation of EFNA3 expression promotes NP cell apoptosis and ECM degradation

**FIGURE 4:**
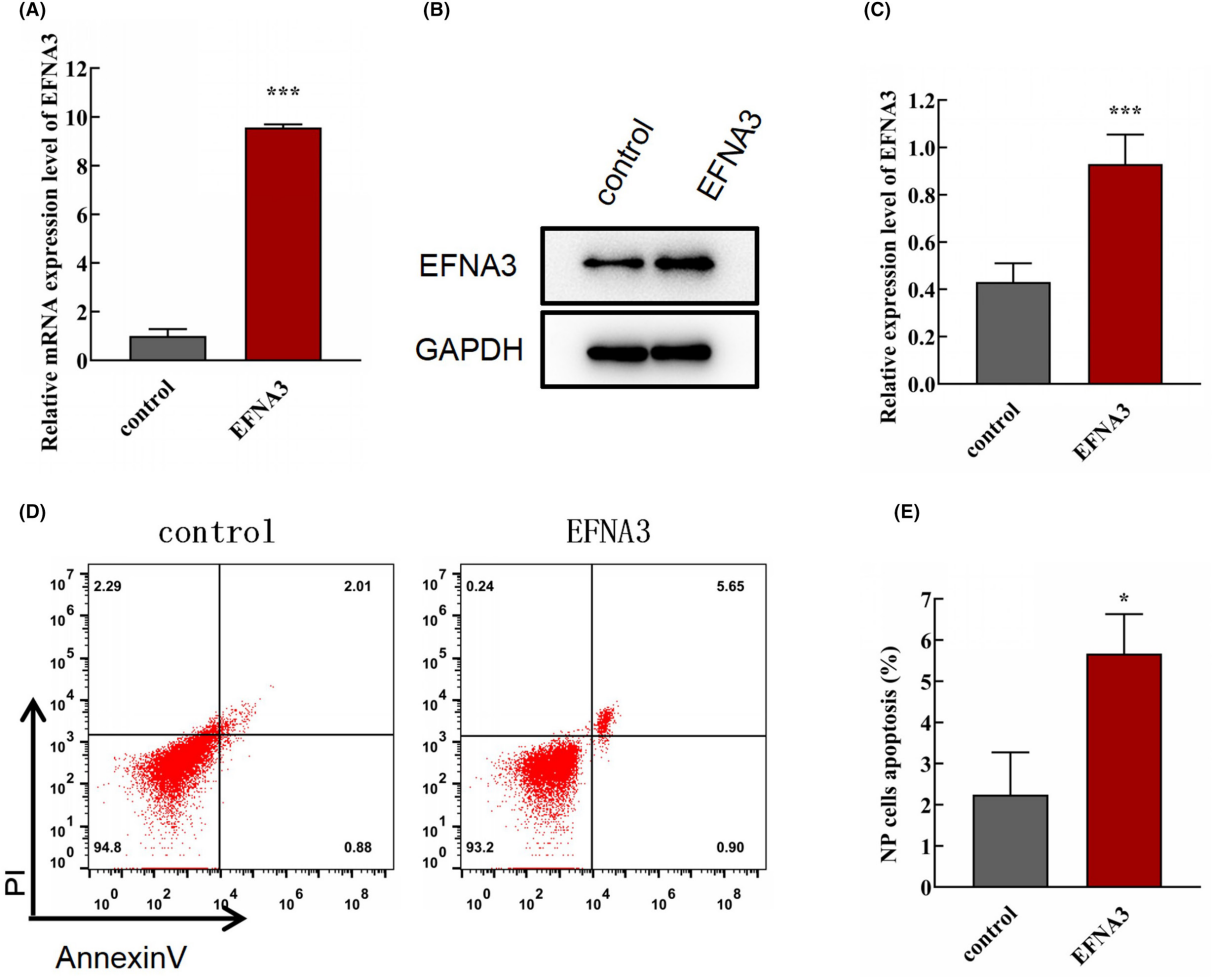
Upregulation of EFNA3 expression promoted NP cell apoptosis. (A) The EFNA3 mRNA expression in NP cells was analysed by qRT‐PCR after transfection with plasmid‐EFNA3 and the corresponding NC (*n* = 3/group, ***p < 0.001, compared with control group). (B, C) The protein expression of EFNA3 in NP cells was analysed by western blotting after transfection with plasmid‐EFNA3 and corresponding NC (*n* = 3/group, ***p < 0.001, compared with control group). (D, E) Assessment of NP cell apoptosis by flow cytometry after transfection with plasmid‐EFNA3 and corresponding NC (*n* = 3/group, *p < 0.05, compared with control group).

**FIGURE 5:**
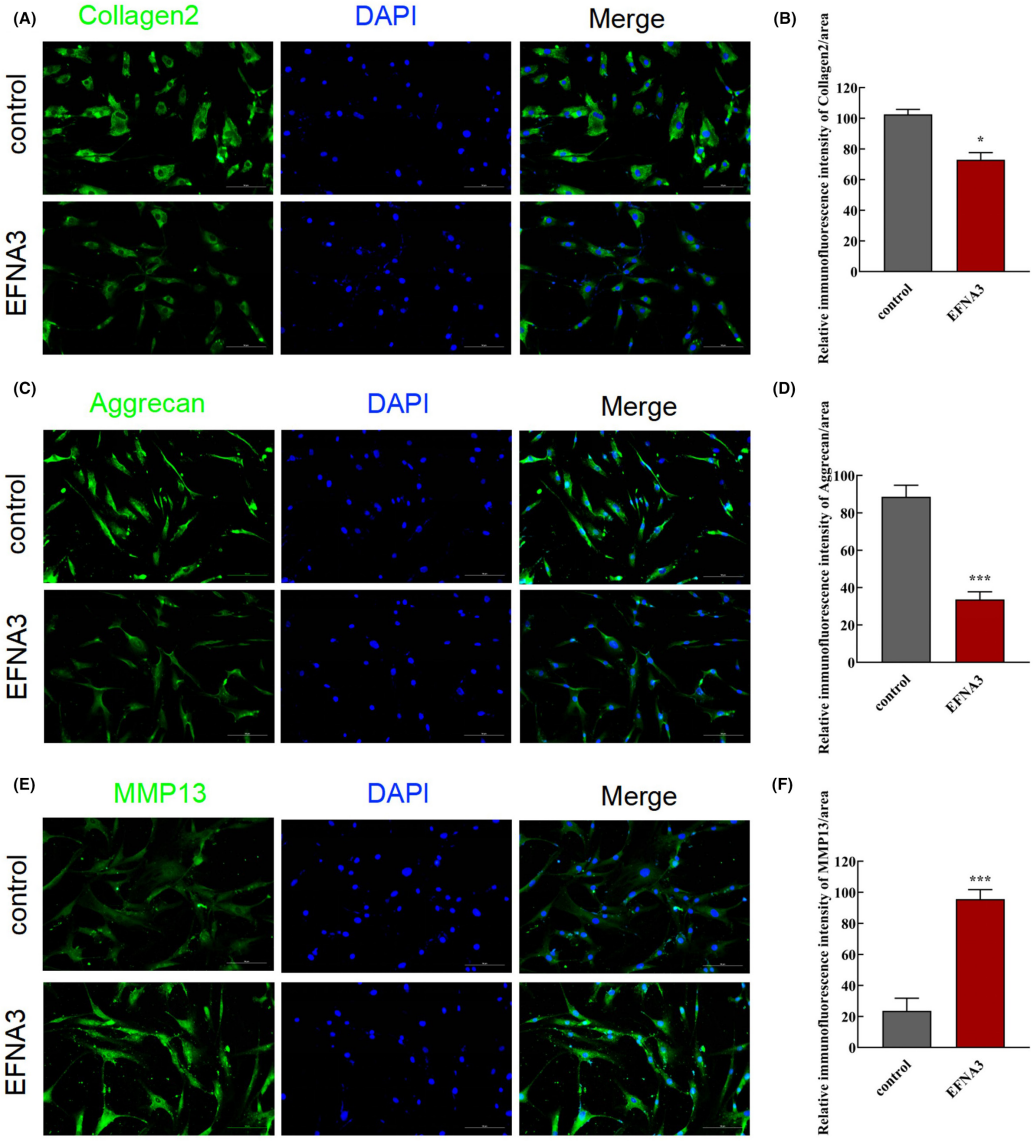
Upregulation of EFNA3 expression promoted ECM degradation. (A–C) The changes of collagen II, aggrecan and MMP‐13 expression were detected by immunofluorescence staining in human NP cells after transfection with plasmid‐EFNA3 and corresponding NC (scale bar = 50 μm). (D–F) Quantification of collagen II, aggrecan and MMP‐13 relative expression in each group (*n* = 3/group, *p < 0.05, ***p < 0.001, compared with control group).

To better understand the possible molecular mechanisms of EFNA3 in modulating NP cells and ECM degradation, EFNA3 was overexpressed in NP cells. As shown in (Figure 4A–C), the expression of EFNA3 was remarkably increased after transfection with plasmid‐EFNA3 at mRNA and protein level (Figure 4A–C, ***p < 0.001, compared with control group), indicating that the plasmid was successfully transfected. Strikingly, flow cytometric analysis demonstrated that NP cell apoptosis was significantly strengthened when EFNA3 expression was upregulated (Figure 4D,E, *p < 0.05, compared with control group), suggesting EFNA3 could facilitate NP cell apoptosis. Additionally, the results of immunocytofluorescence staining showed that EFNA3 overexpression suppressed the expression of collagen II (Figure 5A,B, *p < 0.05, compared with control group) and aggrecan (Figure 5C,D, ***p < 0.001, compared with control group), while promoted the expression of MMP‐13 (Figure 5E,F, ***p < 0.001, compared with control group).

### 3.5 Injection of miR‐365‐NP cells attenuates IDD progression in vivo

**FIGURE 6:**
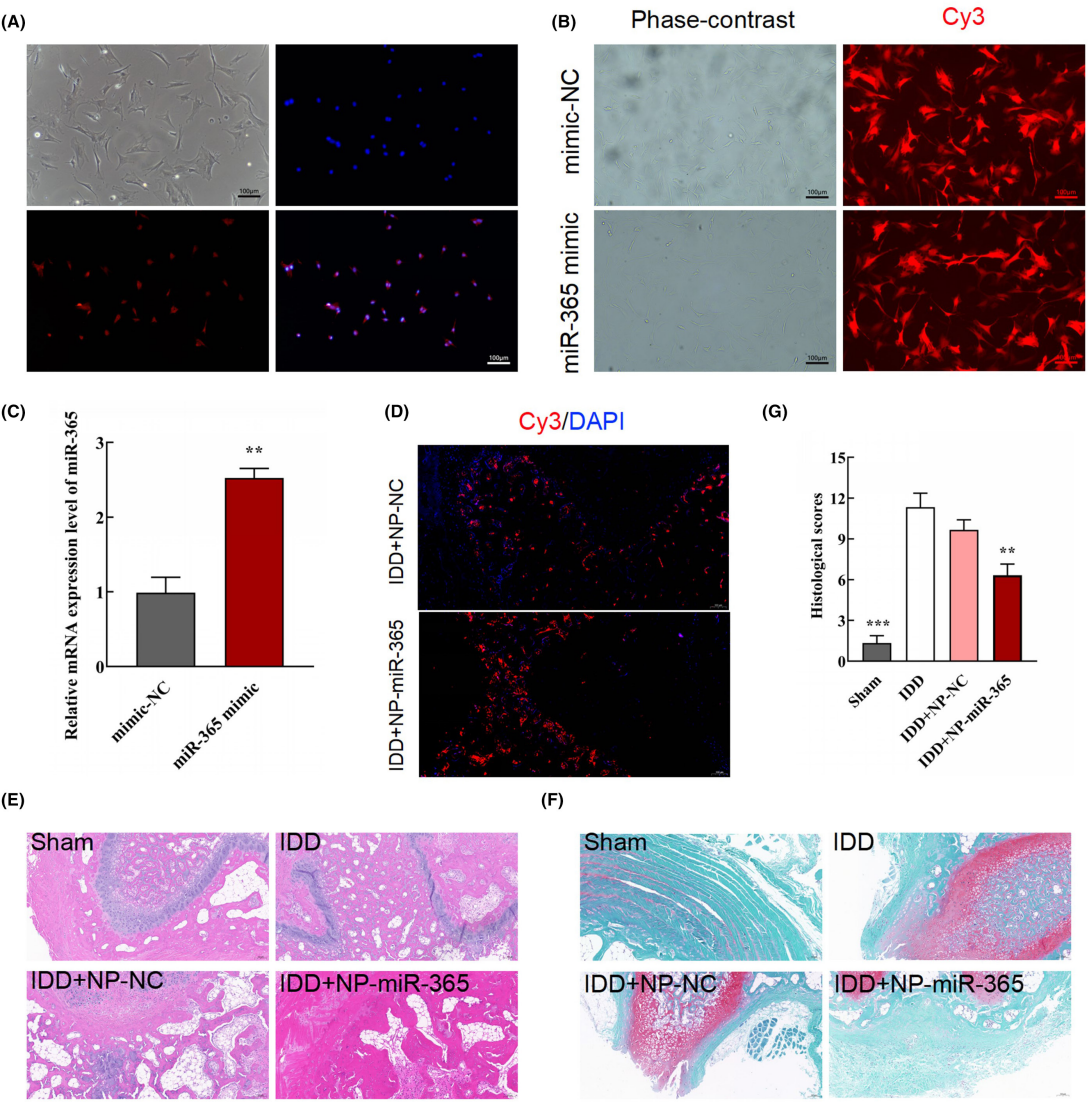
miR‐365 attenuated IDD development in rat models. (A) Characterization and identification of primary NP cells (scale bar = 100 μm). (B) Transfection of primary NP cells used miR‐365 and miR‐NC with Cy3 (scale bar = 100 μm). (C) The relative mRNA expression of miR‐365 was analysed by qRT‐PCR after transfection (*n* = 3/group, **p < 0.01, compared with mimic‐NC group). (D) Immunohistofluorescence staining at 8 weeks post‐IDD (Scale bar = 100 μm). (E) HE staining of rat disc tissue in each group (scale bar = 100 μm). (F) The intervertebral disc degeneration evaluated by Safranin O staining (Scale bar = 100 μm). (G) Histological score in different groups in each group (*n* = 3/group, **p < 0.01, ***p < 0.001, compared with IDD group).

The present results revealed that miR‐365 could attenuate NP cell apoptosis and ECM degradation by directly targeting EFNA3 in vitro. However, the effects of miR‐365 on IDD still remained unclear. In order to further confirm the positive effects of miR‐365 on IDD, we isolated the primary NP cells from rats and injected miR‐365‐NP cells into rats with IDD. Typical NP cells were mostly star‐ or spindle‐shaped and irregularly arranged (Figure 6A). The results of immunocytofluorescence staining of collagen II indicted that NP cells were obtained with high purity (Figure 6A). Both miR‐NC mimic and miR‐365 mimic NP cells were labelled with Cy3 (Figure 6B). Then, the results of qRT‐PCR indicated that the relative expression of miR‐365 was significantly increased after transfection with lentivirus overexpressing Cy3‐miR‐365 at mRNA level (Figure 6C, **p < 0.01, compared with mimic‐NC group).

**FIGURE 7:**
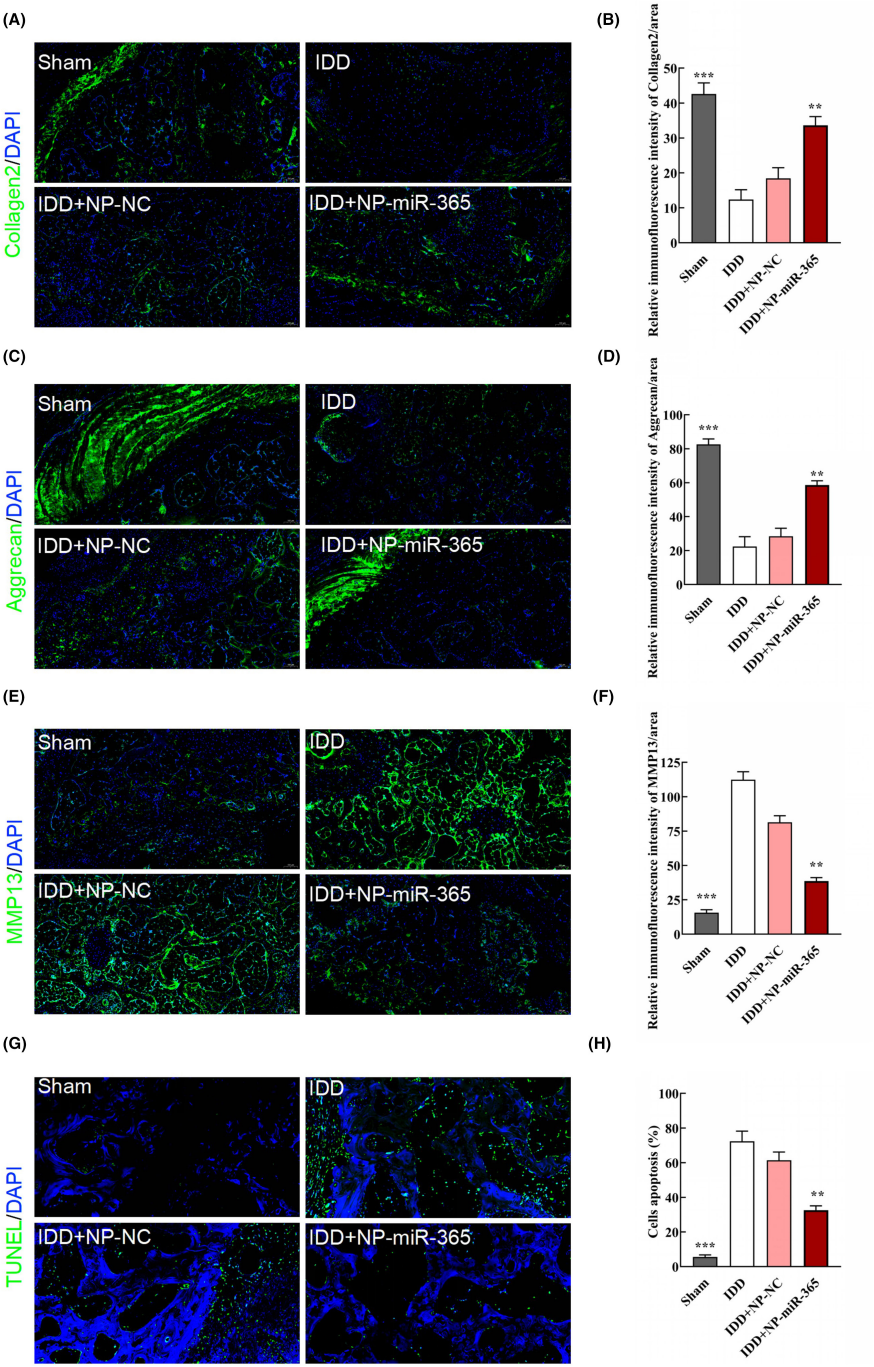
miR‐365 alleviated the ECM degradation and cell apoptosis in vivo. (A,C,E) The expression of collagen II, aggrecan and MMP‐13 were detected by immunofluorescence staining in IDD model treated by NP‐miR‐365 at 8 weeks (scale bar = 100 μm). (B,D,F) Quantification of collagen II, aggrecan and MMP‐13 relative expression in each group (*n* = 3/group, **p < 0.01, ***p < 0.001, compared with IDD group). (G) The apoptotic cells measured by TUNEL staining after 8 weeks (scale bar = 100 μm). (H) Quantification of apoptotic cells in each group (*n* = 3/group, **p < 0.01, ***p < 0.001, compared with IDD group).

At the designed time points, the rats were sacrificed, and the intervertebral disc tissues were harvested to prepare tissue sections. Both in IDD + NP‐NC and IDD + NP‐miR‐365 groups, Cy3‐labelled cells were observed (Figure 6D), suggesting that NP cells were successfully injected into intervertebral disc tissues. Based on the results of HE, the structures of the annulus fibrosus and the NP tissues were clear in sham group, whereas the structures were irregular and obscure in IDD and IDD + NP‐NC groups (Figure 6E). In IDD + NP‐miR‐365 group, the structures of the annulus fibrosus and NP tissues displayed some regularity (Figure 6E). The histological evaluation indicated that miR‐365 alleviated the degeneration of intervertebral disc in IDD rats (Figure 6F,G, **p < 0.01 and ***p < 0.001, compared with IDD group). Likewise, miR‐365 inhibited the expression of MMP13 and enhanced the expression of collagen II and aggrecan (Figure 7A–F, **p < 0.01 and ***p < 0.001, compared with IDD group), implying that miR‐365 may suppress the ECM catabolism. Besides, overexpression miR‐365 remarkably decreased apoptosis in IDD rats treated by NP‐miR‐365 (Figure 7G,H, **p < 0.01 and ***p < 0.001, compared with IDD group).

## 4 DISCUSSION

Currently, compelling findings support the idea that miRNAs are involved in cellular activity by post‐transcriptional regulation.^29^ For instance, miR‐455 was found to modulate TGF‐β signalling and inhibit the SMAD 2/3 pathway, thereby regulating chondrogenesis and degradation in articular cartilage.^30^ Park et al.^31^ discovered that the expression of miR‐127‐5p was significantly downregulated in human degenerative cartilage tissue, leading to cartilage degeneration by regulating the expression of MMP. Various studies have demonstrated that mechanical forces, such as shear stress and cyclic stretch, can influence the expression of miRNAs in different cell lines, and these miRNAs are involved in different cellular responses to mechanical forces.^32^, ^33^ Recent studies have also revealed that dysregulated miRNAs are closely associated with various human diseases, including IDD.^17^, ^29^, ^34^ Although the underlying mechanisms are not completely unravelled, it has been demonstrated that miRNAs exert considerable effects on the development of IDD through regulating inflammatory response, ECM metabolism, NP cell apoptosis and proliferation.^4^, ^17^ In this study, we found that miR‐365 attenuated apoptosis and ECM degradation in human NP cells through EFNA3. In addition, we demonstrated that miR‐365 played a favourable role in modulating cell apoptosis and ECM degradation, thereby ameliorating IDD in rat models.

The manifesting characteristics of IDD are summarized as a reduced NP cell population and ECM loss, which results in a homeostatic imbalance and exacerbates disease progression.^18^, ^21^ Excessive apoptosis predominantly leads to NP cell reduction, which is associated with IDD.^17^, ^35^ The ECM mainly contains collagens and proteoglycans, especially type II collagen and aggrecan, which are indispensable to maintain normal functions in healthy individuals.^36^ Therefore, the synthesis and degradation of ECM keep balanced in healthy intervertebral disc. Once the balance is broken, especially degradation over synthesis, the degenerative disc will occur.^37^ Given that the degradation of ECM is dependent on matrix metalloproteinases (MMPs), for which expression is upregulated in degenerative disc tissues,^38^ it is of importance to seek a more efficient therapeutic strategy for IDD treatment.

miR‐365, as a member of the miRNAs family, plays pivotal role in cell activity and is involved in a variety of human diseases.^23^, ^25^ Kang et al.^23^ reported that miR‐365 inhibits lung cancer cell proliferation by regulating NKX2‐1 expression. Likewise, Nie et al.^24^ also found that overexpression of miR‐365 promoted cell cycle progression and decreased apoptosis of colon cancer cells through targeting Cyclin D1 and Bcl‐2. Similarly, Wang et al.^25^ revealed that upregulation of miR‐365 expression curtailed the proliferation and enhanced the apoptosis of synoviocytes through suppressing IGF1‐mediated PI3K/AKT/mTOR signalling pathway, involving in rheumatoid arthritis (RA). Additionally, recent studies suggest that miR‐365 may impact the progression of IDD through multiple mechanisms.^26^ Specifically, Zheng et al.^26^ demonstrated that miR‐365 induced human endplate chondrocyte degeneration through targeting HDAC4. However, whether dysregulated miR‐365 is associated with IDD still remains unknown.

In the present study, we comprehensively investigated the role of miR‐365 in IDD and identified its corresponding target gene, EFNA3, which was verified through a set of experiments. Specifically, we revealed that overexpression of miR‐365 could effectively inhibit NP cell apoptosis and promote ECM degradation. Collagen II and aggrecan are regarded as the anabolic markers of ECM, whereas the MMP‐13 is the catabolic markers. Strikingly, the upregulation of miR‐365 not only inhibited NP cell apoptosis, but also elevated expression of anabolic markers collagen II and aggrecan. Critically, the catabolic marker MMP‐13 also decreased when expression of miR‐365 was upregulated in NP cells. Conversely, downregulation of miR‐365 expression resulted in the opposite effects on cells apoptosis and expression of ECM anabolic and catabolic markers. Taken together, these results suggested that miR‐365 played a pivotal role in regulating the development of IDD.

To elucidate the targeting gene of miR‐365, we conducted bioinformatic prediction and luciferase reporter assays to substantiate whether EFNA3 is the underlying target of miR‐365. Interestingly, co‐transfection with miR‐365 inhibitor led to an increase in EFNA3 expression. More importantly, overexpression EFNA3 enhanced NP cell apoptosis and ECM degradation, suggesting a critical relationship between miR‐365 and EFNA3 expression. It is increasingly evidenced that EFNA3 acts as a receptor tyrosine kinase ligand, and its expression level has been proved to be aberrant in kinds of cancers.^39^ In addition, overexpression of EFNA3 contributed to promoting tumour cellular activities, including cell proliferation, angiogenesis and invasion.^40^ Hu et al.^41^ showed that miR‐210 could promote angiogenesis and inhibit apoptosis by targeting EFNA3 in a murine model of myocardial infarction. Meanwhile, Wang et al.^42^ demonstrated that enhanced expression of miR‐210 promoted the proliferation and invasion of peripheral nerve sheath tumour cells through partly targeting EFNA3. On the basis of these previous studies, in combination with our findings, we speculated that miR‐365 could modulate NP cell activity by binding to EFNA3. In this study, we found that overexpression of miR‐365 inhibited ECM degradation and decreased MMP‐13 expression, suggesting that miR‐365 likely targeted EFNA3 responsible for cell apoptosis and ECM degradation. More importantly, the data demonstrated that miR‐365‐NP cells injection ameliorated IDD in rats models through reducing cells apoptosis and ECM catabolism. Taken together, these results revealed that dysregulated miR‐365 played a critical role in IDD by targeting EFNA3. Thus, this study may provide a promising therapeutic strategy for the treatment of IDD.

In this study, we reported that overexpression of miR‐365 attenuated the development of IDD by regulating NP cell apoptosis and extracellular matrix degradation in rat IDD models. Further, EFNA3, as the target gene of miR‐365, exerted a crucial role in IDD. However, there are some limitations, which need to be improved and paid attention in future work. On one hand, we only explore the effects of miR‐365 on NP cell apoptosis and ECM degradation. In fact, miR‐365 shares extensive bio‐functions, and it is necessary to further investigate the roles in the progression of IDD. On the other hand, a deeper study including seeking some critical target genes and exploring the signal pathway is required. Although there are still some drawbacks, the present preliminary experiments may lay a foundation for understanding the aetiology and mechanism of IDD, and the results are expected to provide a promising therapeutic strategy for IDD treatment.

## 5 CONCLUSION

In summary, miR‐365 could efficiently alleviate the progression of IDD by modulating NP cell apoptosis and ECM catabolism in rats IDD models. The underlying mechanism is intimately related to EFNA3. Therefore, miR‐365 might be a promising therapeutic target for the treatment of IDD, functioning via EFNA3.

## AUTHOR CONTRIBUTIONS

**Chao Jiang:** Writing – original draft (equal). **Youjun Liu:** Writing – original draft (equal). **Weigong Zhao:** Data curation (equal); formal analysis (equal); software (equal). **Yimin Yang:** Data curation (equal); formal analysis (equal); software (equal). **Zhiwei Ren:** Data curation (equal); formal analysis (equal); software (equal). **Xiaohui Wang:** Formal analysis (equal); software (equal). **Dingjun Hao:** Writing – review and editing (equal). **Heng Du:** Conceptualization (equal). **Si Yin:** Conceptualization (equal).

## FUNDING INFORMATION

This study was funded by the Natural Science Foundation of Shaanxi Province of China (grant number 2020SF‐080 and 2023‐YBSF‐215) and Science and Technology Foundation of Xi'an (grant number 23YXYJ0141).

## CONFLICT OF INTEREST STATEMENT

All authors reviewed the whole manuscript and declared that there were no conflicts of interest.

## Data Availability

The datasets in the present study could be available from the corresponding author on reasonable request.
